# Traditional and Computational Screening of Non-Toxic Peptides and Approaches to Improving Selectivity

**DOI:** 10.3390/ph15030323

**Published:** 2022-03-08

**Authors:** Alberto A. Robles-Loaiza, Edgar A. Pinos-Tamayo, Bruno Mendes, Josselyn A. Ortega-Pila, Carolina Proaño-Bolaños, Fabien Plisson, Cátia Teixeira, Paula Gomes, José R. Almeida

**Affiliations:** 1 Biomolecules Discovery Group, Universidad Regional Amazónica Ikiam, Tena 150150, Ecuador; alberto.robles@est.ikiam.edu.ec (A.A.R.-L.); bruno000mendes@gmail.com (B.M.); josselyn.ortega@est.ikiam.edu.ec (J.A.O.-P.); carolina.proano@ikiam.edu.ec (C.P.-B.); 2 Escuela Superior Politécnica del Litoral, ESPOL, Centro Nacional de Acuicultura e Investigaciones Marinas (CENAIM), Campus Gustavo Galindo Km. 30, 5 Vía Perimetral, Guayaquil 09-01-5863, Ecuador; eapinos@espol.edu.ec; 3 Consejo Nacional de Ciencia y Tecnología, Unidad de Genómica Avanzada, Laboratorio Nacional de Genómica para la Biodiversidad (Langebio), Centro de Investigación Y de Estudios Avanzados del IPN, Irapuato 36824, Mexico; fabien.plisson@cinvestav.mx; 4 Laboratório Associado para a Química Verde-REQUIMTE, Departamento de Química e Bioquímica, Faculdade de Ciências, Universidade do Porto, 4169-007 Porto, Portugal; catia.teixeira@fc.up.pt (C.T.); pgomes@fc.up.pt (P.G.)

**Keywords:** in silico, machine learning, peptides, hemolysis, toxicity

## Abstract

Peptides have positively impacted the pharmaceutical industry as drugs, biomarkers, or diagnostic tools of high therapeutic value. However, only a handful have progressed to the market. Toxicity is one of the main obstacles to translating peptides into clinics. Hemolysis or hemotoxicity, the principal source of toxicity, is a natural or disease-induced event leading to the death of vital red blood cells. Initial screenings for toxicity have been widely evaluated using erythrocytes as the gold standard. More recently, many online databases filled with peptide sequences and their biological meta-data have paved the way toward hemolysis prediction using user-friendly, fast-access machine learning-driven programs. This review details the growing contributions of in silico approaches developed in the last decade for the large-scale prediction of erythrocyte lysis induced by peptides. After an overview of the pharmaceutical landscape of peptide therapeutics, we highlighted the relevance of early hemolysis studies in drug development. We emphasized the computational models and algorithms used to this end in light of historical and recent findings in this promising field. We benchmarked seven predictors using peptides from different data sets, having 7–35 amino acids in length. According to our predictions, the models have scored an accuracy over 50.42% and a minimal Matthew’s correlation coefficient over 0.11. The maximum values for these statistical parameters achieved 100.0% and 1.00, respectively. Finally, strategies for optimizing peptide selectivity were described, as well as prospects for future investigations. The development of in silico predictive approaches to peptide toxicity has just started, but their important contributions clearly demonstrate their potential for peptide science and computer-aided drug design. Methodology refinement and increasing use will motivate the timely and accurate in silico identification of selective, non-toxic peptide therapeutics.

## 1. Peptide Drugs Market and Discovery: A Bird’s Eye View

Peptides are gaining traction on the new drug development agenda, and their number in the clinics grows annually [1]. The hypoglycemic hormone insulin stands out as a pioneering peptide in the medical industry that opened space for the search and applications of these small molecules in the pharmaceutical, diagnostic, cosmeceutical, clinical, and biomedical scope [2]. Since then, in this centenary journey, more than 80 Food and Drug Administration (FDA)-approved peptides constitute an essential portfolio of life-saving molecules. Approximately 19% of these peptide drugs were approved from 2015 to 2019, showing a remarkable advance and success in the last decade [3]. On the other hand, this number increases five-fold when adding peptide-based drugs under clinical trials [4]. About 400 oligopeptides are currently under evaluation offering promising prospects for the era of peptides as clinically viable drugs. An optimistic economic projection also accompanies this considerable growth; as such, the global peptide therapeutics market should expand over the forecast horizon, achieving a compound annual growth rate of 9.4% by 2025 [5]. Currently, this market is a multibillion-dollar industry, valued at US$26.98 billion and projected to double by 2027 [6]. In this context, peptides become a bet with a highly profitable vision. Thus, the market recognizes therapeutic peptides with a renewed and more substantial interest [7].

The chemical nature and properties of peptides, such as versatility, biochemical diversity, and multifunctionality, have guided and motivated the process of identifying new candidates for effective treatments with clinical benefits [8,9]. They offer a potential breakthrough in many diagnostic tools, drug delivery systems, and therapies, including cancer, bacterial infections, parasitic diseases, and others [10,11]. Their small size and structural simplicity do not coincide with their high number of applications and particular characteristics that respond to global needs and the challenging, costly, and complex drug development [12,13]. Bioactive peptides can easily be synthesized and engineered to obtain more druggable versions [14,15]. Their metabolic degradation is predictable and generates non-accumulative compounds for the body. These residual compounds are often associated with less toxicity than many small molecules. However, peptides have limited success in oral administration [16]. Multiple strategies have circumvented this hurdle by using absorption enhancers, enzyme inhibitors, modification by conjugation to polymers or other moieties, encapsulation by liposomes, multiple emulsions, and nanoparticles. These approaches are based on biocompatible systems that enable adequate transport and escalate the routes of administration [17].

Drug development involves a series of steps with complex and important barriers that guarantee the approval of only effective and non-toxic pharmaceuticals [18]. Candidate molecules face a series of challenges, and selectivity is one of the main pillars for their progression into the clinics. Despite the many candidates constantly discovered, only a tiny fraction is converted into affordable, scalable, and effective therapies [19]. Drug toxicity remains a latent issue [20], and peptides are no exception to this rule [21]. Although databases reveal more than 10,000 unique bioactive peptide sequences [22,23], less than 1% of this group became FDA-approved drugs. Several bioactive peptides have shown toxicity, especially their disruptive action on red blood cells (RBCs) [24]. Therefore, toxicity to healthy eukaryotic cells remains a major bottleneck in the approval rate of new pharmaceutical peptides [25].

**Figure 1:**
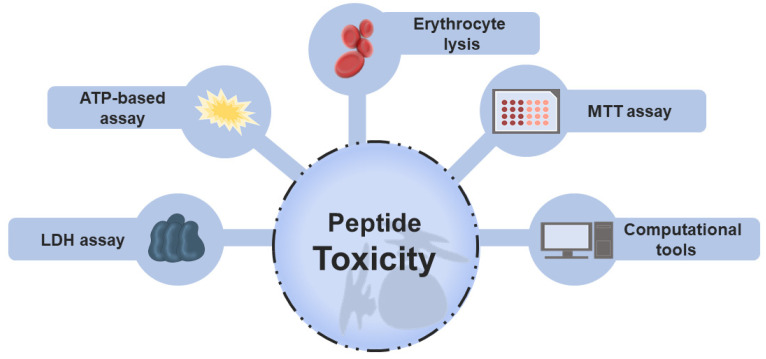
Main approaches employed to evaluate the toxicity of peptides. Traditional screening of non-toxic peptides is performed using different in vitro techniques, including MTT, LDH, erythrocyte lysis, and ATP-based assays. These methods are based on the measurement of intracellular markers released during cell death or lysis, such as hemoglobin (red blood assay), enzymes (LDH assay), or on the analysis of cell viability determined by enzymatic activity, measured, for example, by the MTT assay or by the amount of cell energy (ATP-based assay). Recently, computational models were reported to assist in peptide toxicity prediction.

Toxic peptides are categorized into three main groups: cytotoxic (general), hemolytic (toxic to RBCs), and immunotoxic (modulate the immune response in an undesirable manner) peptides [26]. Toxicity evaluation is naturally of extreme relevance in drug development and approval [27], and different wet-laboratory approaches have been used to this end. Historically, in vitro assays have been pivotal to analyzing the toxic activity in peptides research. Hemolysis assay, lactate dehydrogenase (LDH) release assay, colorimetric 3-[4,5-dimethylthiazol-2-yl]-2,5 diphenyl tetrazolium bromide (MTT) assay, and ATP-based assay are the experimental protocols most frequently used to measure toxicity [28,29]. In general terms, these methods are based on intracellular biomarkers such as LDH and hemoglobin released when there is cell damage or cell viability, determined by enzymatic activity or quantification of the energy currency, ATP. Figure 1 highlights these in vitro assays that provide insights into the selectivity and safety of peptides.

Computational tools have revolutionized at a tremendous speed the chemical, biological and pharmaceutical fields, including peptide science [30,31]. In the last decade, the development of such predictive tools has permitted the discovery of novel toxic and non-toxic peptides as well as the design of analogs with reduced toxicity [32]. Recent machine learning (ML)-driven methods [33,34,35,36,37,38,39] predicting the peptide hemolytic action are described in this review. Such methods are considered cost-effective and time-saving strategies to support the development of peptide-based drugs. Current predictors are limited and possibly biased by the number of peptide sequences, their diversity, and the associated biological data [40]. The in silico predictive technologies of the cytotoxic action of peptides are still in their infancy but have offered ample opportunities that reduce the number of expensive failures. This work first discusses the primary cell model used to study the toxicity of peptide candidates and how new data-driven computational methods have been crucial to understanding structure-activity relationships (SAR) and contributing to the selection of possible safe peptide templates for synthesis and evaluation.

Many bibliographic studies have covered the therapeutic effects of peptides in the most diverse areas [41,42,43,44], including the discussion of market trends, current challenges, and prospects [1,5,45]. On the other hand, several reviews have recently highlighted the development of in silico methods to support the discovery of antimicrobial peptides [46,47,48]. However, to our knowledge, the computational advances in the field of peptide toxicity, although extremely important, have not been thoroughly addressed yet. Therefore, here, we focused on the in silico frameworks that speed the discovery and design of non-toxic peptides. We started by documenting the applicability of the standard hemolysis assay in initial hit screening. We then described reported predictive models for hemolytic activity. This review integrates the recent computational advances that support the identification, design, and synthesis of non-toxic peptides with greater probabilities of clinical translation. Lastly, we discussed possible directions and perspectives on how computational advances should shape the future of peptide-based drugs and the multidisciplinary drug development process. The most frequent approaches to increasing the selectivity and safety of therapeutic peptides are also reviewed.

## 2. Red Blood Cells as a Standard Model for Toxicity Assessment of Peptides

For a new peptide to be considered a promising therapeutic candidate, it must have minimal cytotoxic effects on healthy host cells [49]. The value of in vitro studies for toxicity prediction is very high since it allows obtaining baseline information on the harmful effects, allowing better administration and directing of resources to other study stages [50,51]. In other words, the in vitro toxicity assessment of a natural [52] or a synthetic compound [53] is the first step to be carried out in order to consider new pharmaceutical formulations at the in vivo scale and toward clinical trials [54].

As previously mentioned, there are several methodologies for determining the in vitro toxicity of a compound. Although the activities or characteristics obtained vary between methods, they basically are indicators of membrane rupture and cell death [45,55,56,57]. Temporal analysis, data interpretation, and sensitivity are other important parameters that vary between techniques, including bioactive peptides studies [55]. Given this heterogeneity, there is no universal answer as to which is the most effective methodology. Some parameters are more advantageous than others, and their use will depend on different conditions (resources, time, and reproducibility). The aforementioned in vitro assays also have shortcomings that must be addressed before selecting the most suitable one(s) according to the initial objectives. Generally, the optimal technique presents high sensitivity, simple reproducibility, rapid results generation, and is cost-effective [58]. For this reason, hemolysis assay has been the reference protocol for the early toxicity screening phase [59].

Erythrocytes are the most abundant and crucial cells in the circulatory system, given their vital oxygen-carrying function. They are very particular cells, as they lack a nucleus and organelles with a membrane inside [60]. One can put into question the validity of their use as a standard and valuable in vitro model for cytotoxicity assessment [61], which is justified by their abundance in organisms, easy cultivation, and availability in significant amounts [54]. The isolation and cultivation of other cell types often present higher complexity, i.e., rat intestinal epithelial cells and human umbilical vein endothelial cells [62] or the use of mice-derived macrophages [9]. In addition, one can take advantage of the lack of internal membrane structures in red blood cells (RBCs), which can facilitate the standardization and interpretation of results. In fact, despite normal nucleated cells not being comparable to the structural simplicity of RBCs, these can experience morphological and quantitative alterations that reflect significant damage and can act as a predictive marker for the toxic impact of test compounds [59,63,64]. These features make the RBCs a cheap, fast, and effective template for evaluating toxicity.

Hemolysis is a natural or disease-induced event potentially caused by novel small molecule drug candidates or cationic peptides [65,66,67]. Briefly, hemolytic assays determine the disruption and destruction of RBCs and have been promoted not only due to their simplicity but also because of the structural and biochemical composition similarities between RBC membranes and those of other human cells [68]. In percentage terms, the erythrocyte membrane is made up mostly of proteins (39.5%), followed by lipids (35.1%), water (19.5%), and carbohydrates (5.8%) [59]. The interplay between their components, lipid composition, and high oxygen tension makes RBC membranes ideal models for studying disturbances caused by oxidative stress and induced by an external molecule [63].

Human-derived RBCs are the first line of use; however, some studies have used erythrocytes from other animals, such as cows [55], sheep [69], rats [70], pigs [71], dogs [29], and rabbits [72]. Dennison and Phoenix [73] have demonstrated that the hemolysis produced by the Modelin-5-CONH_2_ peptide (300 µM) was 12% for sheep RBCs and 2% for those from humans and pigs. This observation has been attributed to the differences in the contents of phosphatidylcholine and sphingomyelin in the erythrocytes membranes. In general lines, sheep RBCs have a lower percentage of phosphatidylcholine and a higher percentage of sphingomyelin than human and porcine RBCs, demonstrating that those components can be key mediators for the hemolytic action of the peptide. In agreement with this investigation, Greco et al. [29] measured the activity of 24 synthetic peptides on RBCs of dogs, humans, rats, and cows. They noted heterogeneous reactions among these species’ cells, being those from dogs the most susceptible. In addition to interspecies variations in erythrocyte membrane components and their organization, there are also dissimilarities in the abundance of ion channels and aquaporins, resulting in differences in tolerance to sudden changes in permeability and osmotic balance [74,75].

The general hemolytic assay procedure is based on the exposure of the erythrocytes to a specific agent (in this case, peptides) at a selected range of concentrations for the subsequent spectrophotometric quantitation of released hemoglobin at a given wavelength [76]. Usually, measurements are performed at 405 [29], 414 [28], 450 [77], 540 [54], and 576 nm [73], among other wavelengths, taking advantage that the levels of hemoglobin are directly proportional to the number of RBCs that have been lysed. The absorption values obtained for the negative (0% hemolysis) and positive (100% hemolysis) controls are taken as a reference to calculate the percentages of hemolysis, which are analyzed and compared using standard statistical tests, e.g., one-way ANOVA and Tukey’s test [9], *t*-test [78], Boltzmann sigmoidal equation [79], Mann–Whitney U-test [80], and others. Generally, the percentage of hemolysis at a given concentration is obtained by the following equation [28,77]:

(1) $$\;\%\;\mathrm{Hemolysis}=\frac{\left(\mathrm{Abs}\;\mathrm{sample}\;-\;\mathrm{Abs}\;\mathrm{negative}\;\mathrm{control}\right)}{\left(\mathrm{Abs}\;\mathrm{positive}\;\mathrm{control}\;-\;\mathrm{Abs}\;\mathrm{sample}\right)}\times 100\;$$

where Abs represents absorbance. In some studies, there are minimal variations to this formula [29,53,81,82].

Triton X-100 and melittin are the most recognized positive controls [28,53,83]. Melittin is a cationic 26-mer membrane-binding polypeptide very abundant in *Apis mellifera* venom proteome. This amphipathic molecule with a basic *C*-terminal region induces a high lytic activity on a large number of cell types and at very low concentrations [84]. However, its chemical synthesis and purification may represent an extra cost or time if the laboratory does not have the required equipment. Triton X-100 is often used as an alternative positive control for hemolysis. This nonionic membrane-damaging detergent is cheap, commercially available in high purity, and does not interfere with the spectrophotometric measurements [28].

**Figure 2:**
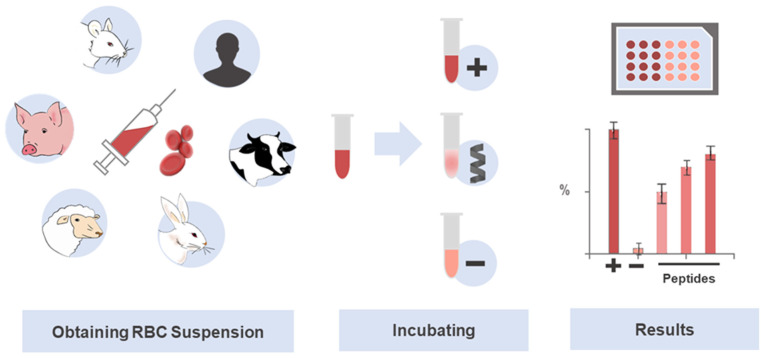
Use of erythrocytes in the investigation of selective and potentially translational therapeutic peptides. The hemolysis assay is a standard technique widely used in toxicity screening of drug candidates, especially peptides. The abundance and easy obtainment of RBCs, together with the simplicity of the experiment, contribute to its prioritization in toxicity studies. The RBCs lysis protocol involves a colorimetric assay, which determines the amount of hemoglobin released after peptide-induced cell damage. Serial dilutions of the peptides are first prepared in parallel with the RBCs suspension, which is obtained by centrifugation and dilution. Then, the peptides, positive, negative, and, eventually, other controls are incubated with the RBCs solution to deliver the raw data that is next analyzed and translated into an HC50.

The parameter to express the toxicity toward erythrocytes is known as the concentration causing 50% of hemolysis, or HC_50_ [85]. This point of reference has been assessed in many studies on peptides, with low values pinpointing greater hemolytic activity, whereas high ones indicate lower toxic activity of the peptides. HC_50_ values are highly variable, with some extremely toxic peptides showing very low values in the nM or low μM range, e.g., 1.7 µM for Melittin [86], 2.9 µM for MG-H1 [87], or 600 nM for PGLa [77]; other peptides show no hemolytic action at high concentrations (≥100 mM), e.g., Dermaseptin S1 (>100 µM) [88], Ranatuerin-1 (140 µM), MP (100 µM) [89], Modelin-5 (300 µM) [73], Ranatuerin-2ARa (100 µM), Esculentin-1ARa (120 µM) and Palustrin-3AR (200 µM) [90,91], Ascaphin-8 (115 µM) and [K^19^] ascaphin-8 (>800 µM) [92]. Figure 2 illustrates the general hemolysis assay protocol as a practical means of screening peptide toxicity.

Some studies take the selectivity index (SI) as the most suitable indicator for drug safety, as it broadens the vision by giving a two-dimensional aspect that integrates therapeutic and toxic components. The SI is the ratio between the concentration that is toxic to 50% of the reference host healthy cells in a cytotoxicity assay (HC_50_ in the case of hemolysis assays) and the concentration that causes the desired therapeutic action on the target cells (e.g., concentration causing growth inhibition on 50% of pathogen cells, or IC_50_). Hence, the SI reflects the therapeutic window between toxicity and biological effect [93]. High values drive the next steps in evaluating the test drug, e.g., bioactive peptides. Some examples of antimicrobial peptides with high SI are presented in Table 1.

A significant rule or consensus has not been established yet in the structural determinants underlying peptides’ hemolytic activity [28]. This understanding is complex, requiring multidisciplinary efforts. The clues collected to date point to a preponderant role of peptide charge, amphipathicity, and hydrophobicity [91,101], contributing to the stabilization of an amphipathic secondary structure [102]. Of the 20 natural amino acids, tryptophan has been touted as a critical residue since its presence in a given peptide mediates molecular interactions with cholesterol present in mammalian membranes and, consequently, their disturbance or even disruption [103,104,105]. On the other hand, positively charged amino acid residues such as arginine and lysine have also been shown to influence RBCs membrane damage. Dathe et al. [106] evaluated the activity of analogs of magainin-II with variable charge, demonstrating that the increase in this parameter (+5) enhances the antimicrobial activity. However, this must be carefully analyzed when generating peptide analogs so that modifications to greater interaction with pathogenic targets do not increase lytic effects on host cells.

The basis of the hemolytic assay allows the evaluation of several peptide concentrations simultaneously, reducing time and costs, as well as enabling easy reproducibility and a significant reduction in the use of in vivo models in concordance with bioethical concerns in animal experimentation [58]. Notably, the human RBCs are a valuable and efficient cellular model to obtain a rapid in vitro approximation of the toxic damages of a peptide in the body, as well as to uncover patterns and (cellular/molecular) mechanisms that can directly influence peptide action [107,108]. An example of this is the study carried out by Ahmad et al. [109], in which the antibacterial and hemolytic activity of a peptide derived from the “leucine zipper” structural motif coined LZP and six analogs were analyzed. The native LZP peptide induced the highest percentage of hemolysis in the 0–30 µM range. The analogs, which possessed replacements of leucine by alanine residues at specific positions, had significantly reduced hemolytic activity; namely, the LZP (L8A/L11A), LZP (L4A/L8A), and LZP (L4A/L11A) analogs induced a percentage of hemolysis close to 0% in the same concentration range. Antibacterial activity of the native peptide and all its analogs did not change significantly, remaining in the 5.6–7.8 µM range against different bacteria.

## 3. Computational Tools and Databases for Hemolytic Activity Prediction

The expanding amount of available information on different peptide structures and their effects has made it possible to develop in silico prediction models on the hemolytic activity of a peptide, highlighting the most influential amino acids. For instance, Langham et al. [110] investigated the quantitative structure-activity relationships (QSAR) underlying the selectivity of five protegrin-like AMPs based on their main physicochemical properties. In brief, the authors demonstrated a strong correlation between the length, mean number of acceptors, and energy term of the β-hairpin peptides and their toxicities. Experimental analysis of toxicity revealed that the model accurately identified the most and least toxic peptide. The advent of these modern computational techniques is adding a further dimension and alternative to toxicity testing panels. These emerging tools effectively analyze multidimensional data, may recognize patterns, and formulate reasonable conjectures [111], generating a new paradigm for the early stages of peptide drug development.

**Figure 3:**
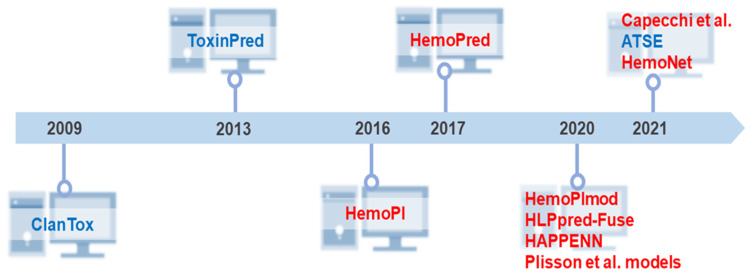
Historical overview of the development of freely available tools and models for prediction of peptide toxicity. Big biomedical peptide data have been explored to design new predictive methods that facilitate adequate access to the full potential of peptides. Despite the many years of peptide science, our literature review demonstrates that these in silico approaches are relatively new. From the pioneering and innovative ClanTox [112] and ToxinPred [40] launched in 2009 and 2013, respectively, ten high-throughput computer toxicity prediction tools were developed that mainly predict peptides’ hemolytic effects. Most of them have been released in the last 5 years. Capecchi et al. [38] and HemoNet [113] are the latest hemolytic classifiers. Some predictors such as HAPPENN [36] and HemoPI [26] have more than one version. HemoPI has 5 SVM methods, while HAPPENN is composed of 3 methods. However, due to the difference in performance reported by the authors, for this chronology, we considered only the best-in-class performance methods. The three Plisson models [37] were considered due to high similarity in performance metrics. Peptide toxicity predictors are highlighted in blue, and the classifiers for predicting peptides’ hemolytic activity are colored in red.

Two seminal approaches preceded the current computational tools for unraveling the hemolytic activity of peptides. The first one occurred in 2009 when Naamati et al. developed the first classification model to find out whether or not animal proteins could be toxic [112]. The second milestone occurred in 2013 when Gupta et al. created ToxinPred as the first web server to estimate the toxicity of peptides [40]. Thereafter, the focus and development of toxicity prediction models were directed at the peptide hemolytic capacity. In 2016, Chaudhary et al. developed the first hemolytic peptide classifier called HemoPI (https://webs.iiitd.edu.in/raghava/hemopi/ (accessed on 25 January 2022)) [26]. The following year, Win et al. developed HemoPred (http://codes.bio/hemopred/ (accessed on 25 January 2022)) [33]. However, the rise of machine learning-guided prediction of peptide toxicity to RBCs occurred only in 2020, when three novel methods became available on the web for such purpose: HemoPImod (https://webs.iiitd.edu.in/raghava/hemopimod/ (accessed on 25 January 2022)) [34], HLPpred-Fuse (http://thegleelab.org/HLPpred-Fuse/ (accessed on 25 January 2022)) [35], and HAPPENN (https://research.timmons.eu/happenn (accessed on 25 January 2022)) [36]. In addition to these, also in 2020, Plisson and co-workers developed an open-access program in Python and compared different machine learning algorithms in which three models stood out as the optimal prediction of hemolytic activity [37]. The novelty of this study lies in establishing the applicability domain of hemolytic models using multivariate outlier detectors. In 2021, Capecchi et al. created another in silico model to elucidate the lytic capacity of peptides on blood cells [38], Yaseen et al. developed the most recent hemolysis model, which considers *N/C*-terminal modifications and L or D amino acids in a primary sequence [113]. In the same year, a web server was established based on a new peptide toxicity predictor named ATSE. Figure 3 summarizes this chronology, comprising 10 different algorithms to predict peptides’ hemolytic activity.

The emergence of peptide toxicity predictive programs is probably due to: (i) the ease of access to high-performance computers to process biological information; (ii) a better scope and understanding of peptide structural and functional characteristics; and (iii) an increase in the elaboration of databases of both AMPs and hemolytic peptides.

Concerning the second point (ii), it is notorious how the number of parameters used in predictive algorithms has increased dramatically from 2013 to 2020. For example, ToxinPred only uses the composition of amino acids, dipeptides, and structural motifs as predictors [40], while HAPPENN makes further use of several physicochemical descriptors to classify peptides [36]. The descriptors that some of the most current prediction programs take into account for prediction of peptides’ hemolytic effects are: length, molecular weight, charge, charge density, isoelectric point, instability index, aromaticity index [114], aliphatic index [115], Boman index [116], hydrophobicity [117,118,119], the Amino Acid Selectivity Index Scale (AASI) for helical AMPs [120], the Acidic, Basic, Hydrophobic, Polar, Aromatic, Kink-inducer (ABHPRK) features scale [121], side chain bulkiness [122], amino acid charges, the COUGAR selection of peptide’s descriptors in the ModlAMP’s Python package (https://modlamp.org/, accessed on 25 January 2022) [121], the energy of insertion of amino acid side chains into lipid bilayers (Ez) [123], side chain flexibility [124], polarity [125], the Isotropic Surface Area and Electronic Charge Index (ISAECI) of amino acid side chains [126], α-helix propensity [127], the MSS topological shape and size parameter for amino acid side chains [128], the MSW amino acid scale based on a principal component analysis (PCA) of the molecular surface-based Weighted Holistic Invariant Molecular (WHIM) descriptor [129], the pharmacophoric feature scale, pepArc, based on hydrophobicity, polarity, charge, and presence of proline residues [121], the PPCALI scale that is derived from PCA of over 140 amino acid property scales [130], refractivity [131], t scale [132], transmembrane propensity [133], and the z3 (electronic properties) [134] and z5 (electronegativity, heat of formation, electrophilicity and hardness) [135] Z-scales for amino acids in peptide sequences. The packages that have been used in Python to establish these physicochemical descriptors are modlAMP [121], ChemoPy [136], and RDKit [137].

As for the last point (iii), over 10 databases (DBs) of AMPs have been made publicly available on the web. These DBs are compiled in Table 2, along with four examples of DBs of hemolytic/toxic peptides (HLPs) that have been developed to set forth toxicity data. The first of these four DBs (https://webs.iiitd.edu.in/raghava/toxinpred/dataset.php (accessed on 25 January 2022)) was created during the development of the ToxinPred server and focuses on describing toxicity in general [40]. It was developed using toxic proteins/peptides obtained from different databases. The second DB, Hemolytik (http://crdd.osdd.net/raghava/hemolytik/ (accessed on 25 January 2022)), focuses on peptides’ hemolytic activity [138]. The HemoPiMOD DB also details hemolytic activity (https://webs.iiitd.edu.in/raghava/hemopimod/download.php (accessed on 25 January 2022)) but focuses on modified peptides instead [34]. The DBAASP-Hemo DB is a subset of peptides from the Database of Antimicrobial Activity and Structure of Peptides (DBAASP; https://dbaasp.org/ (accessed on 25 January 2022)) whose hemolytic activity is also displayed [38]. It should be noted that five (HemoPI, HemoPred, HLP-pred-Fuse, HAPPENN, and Plisson models) out of the eight algorithms thus far developed to predict peptides hemolytic action took data from the Hemolytik DB and were subjected to the criteria set by Chaudhary et al. [26].

## 4. Scheme and Scope of Hemolytic Classifiers

To generate an in silico model to unravel the hemolytic activity of peptides, researchers generally focus on the following stages: (1) pre-processing of the data sets chosen for the classification models; (2) sampling and preparation of training and test data sets; (3) development, selection, and validation of predictive models; (4.) application of predictive models. Although generalist, these stages are shared by most modern computational tools with some modifications.

In the first stage, the developers select the peptide sequences from online DBs that they are going to use to build their supervised models. To date, the eight aforementioned models have been using one of the following peptide libraries; Hemolytik [26,33,35,37,113], HemoPiMOD [34] or DBAASP-Hemo [36,38,113]. Each sequence is then labeled with the property or activity of interest (e.g., hemolytic or non-hemolytic). For example, a binary classifier has two classes where a value of 1 indicates hemolytic peptides, whereas a value of 0 stands for their non-hemolytic counterparts or vice-versa. Of note, most hemolytic predictors were developed using (binary) classification algorithms and not regressions due to the discrepancy in biological data (e.g., HC_50_) from the many research laboratories. In general, QSAR models such as these hemolytic predictors are based on the hypothesis that there is a mathematical relationship between the biological activity or property (e.g., hemolytic activity) and the diversity of bioactive peptide sequences. The developers will then identify and measure a series of features/variables that approximate the differences, such as various local or global physicochemical descriptors, amino acid compositions (i.e., single residues, k-mers, etc.), atomic composition, or other structural motifs [26,33,34,35,36,37,38,113]. These features or variables can be easily accessed once the peptide sequences are encoded using different programs in Python [34,35,36,37,38,113], Motif—EmeRging and with Classes—Identification (MERCI) [26] and indirectly with Simplified Molecular-input Line-entry System (SMILES) and RDKit or modlamp packages [34]. The last step in data pre-processing requires removing duplicated information or missing values and data normalization. For example, in HAPPENN, data reduction was employed when the sequence similarity was higher than 90%, according to the CD-hit software [36]. In the development of the Plisson models, the databases associated with HemoPI-1, HemoPI-2, and HemoPI-3 were cleaned of missing data, duplicates, and later normalized [37]. Finally, many classification models must take into consideration balancing the distribution of their classes, using sampling methods to avoid possible sampling bias. The Capecchi model used 2907 inactive peptide sequences to balance the classes, where 1453 were designed based on the same data subset length distribution, and 1454 were randomly generated sequences [38].

During the second stage; data preparation, the developers divide the labeled peptide data set into two subsets; the main model/training set (75–90% of the whole data set) used for model building and one smaller data set (25–10%) used as external validation [152]. To minimize the risks of overfitting, the model set is often subjected to cross-validation. For instance, in ten-fold cross-validation, sequences are randomly divided into 10 subsets (folds): 9 sets train the models, and the remaining set is the internal testing set.

For the third stage, building models, researchers explored several classification algorithms that best fit the features (e.g., physicochemical descriptors) with the classes (e.g., 1: hemolytic or 0: non-hemolytic peptide). The hemolytic models have actually used the following (binary) classification algorithms; support vector machine (SVM) [26,33,35,36,37,38,40,113], Naïve Bayes (NB) [38], K-nearest neighbor (KNN) [26], multilayer perceptron (MLP) [26], logistic regression (logit) [26,37], J48 [26,33], random forests (RF) [26,33,35,36,37,38,40,113], simulated (SNN) and recurrent (RNN) neural networks (NN) [36,38,113], k-nearest neighbor (k-NN) [34,35,37], extremely randomized trees (ERT) [34], ridge regression (RR) [34], gradient boosting (GBoost) [35,37], adaptive boosting (AdaBoost) [35,37], linear (LDA) and quadratic (QDA) discriminant analysis [37], classification and regression trees (CART) [37], and extreme gradient boosting (XGBoost) [37,113]. Each classification model is evaluated using a series of performance metrics. Among the most used metrics are: accuracy, precision, recovery, Cohen’s Kappa coefficient, Matthew’s correlation coefficient (MCC), and the area under the receiver operating characteristic curve (AUC-ROC). The models with the best performance metrics between the model and validation sets will be selected. Eventually, the models will be optimized by tuning their respective hyperparameters. Table 3 details the best peptide predictive models for hemolytic activity using accuracy and MCC as points of comparison. A high-quality data set is a central element of extreme importance for establishing reliable hemolytic peptide prediction models. In this context, some details of data sets used to develop and evaluate the methods for predicting toxicity peptide are also shown in Table 3.

In the last stage, the best models are applied against an unlabeled external library of natural peptides or randomly generated sequences (testing set) to predict their hemolytic activity. Usually, peptides are divided into classes (0: non-hemolytic and 1: hemolytic peptide). Additionally, a class probability *p* is assigned, which reflects the chance to belong to a given class (probabilistic prediction values can range from 0.00 to 1.00). These scores are converted into binary classification values employing a threshold, such as 0.5. Thus, for example, the three models developed by Plisson (GBoost, LDA, and XGBoost) were later applied to the APD3 database. The predictive results showed that ≈70% of the 3081 natural peptides evaluated are able to induce hemolysis [37]. On the other hand, Capecchi et al. used their adjusted generative models to sample 50,000 amino acid sequences. Non-hemolytic peptides with antibacterial activity were filtered. In summary, 3046 peptides were considered as antimicrobial agents against Gram-negative bacteria, while 2717 sequences were predicted to be active against Gram-positive [38].

A total of 8 of the 10 machine learning models can predict hemolysis for natural peptide sequences. HemoPiMod classifies the hemolytic activity of chemically modified peptide sequences [34], and HemoNet predicts the hemolytic action by peptides that include modifications in the C/N termini or L and D amino acids [113]. Classification models have a minimum accuracy of 76% and a minimum MCC of 0.52. The highest values of Acc and MCC achieved 98.4% and 0.97, respectively. These results emphasize a high predictive power useful in the drug discovery process. Relevantly, despite addressing general toxicity, i.e., not explicitly targeted at RBCs, ToxinPred and ATSE were included in Table 3 due to their historical value to other models for peptide hemolytic activity [39,40].

All these predictive models are easily accessible and user-friendly, even for non-experts in artificial intelligence with the development of web servers (API). The user does not need to have programming skills to run several peptide sequences simultaneously. Their responses to a query FASTA could be obtained within seconds. Some of these servers only allow one query (one peptide sequence) at a time. The more recent predictors such as HemoPred [33], HLPpred-Fuse [35], and the programs created by Plisson et al. [37], Yaseen et al. [113], and Capecchi et al. [38] perform the predictions against sizable libraries. The latter examples require Python skills to predict multiple sequences.

In principle, most developers of these computational strategies only validated their generative models through in silico approaches using statistical parameters calculated based on reliable data sets that include experimental data from hemolytic and non-hemolytic peptides previously characterized. The investigation performed by Capecchi and collaborators is an exception [38], which also contains experimental verification. In this study, a combination of supervised and unsupervised learning aided the selection of a library of peptide sequences of maximum of 15 residues for chemical synthesis. The evaluation of the hemolytic and antimicrobial effects of such short peptides reaffirmed the potential of virtual strategies to guide the discovery of non-toxic antibiotic candidates. In line with experimental validation, researchers have incorporated modern in silico strategies into their workflow [38]. Next, we review some examples.

Mnif and collaborators evaluated through in silico and in vitro approaches the hemolytic properties of a 19-mer cell-penetrating peptide with antibacterial activity against *Staphylococcus epidermidis.* The hemolysis analysis confirmed the non-toxicity suggested by HemoPred online software [153]. Similarly, the hemolytic results of cecropins are in close agreement with previous conclusions from this web server [154]. In silico screening of the properties of peptides from the genome of *Lactobacillus casei* HZ1, including bioinformatics analysis by HemoPI, allowed the identification of a highly active AMP against *S. aureus* [155] and an anticancer sequence [156], both with low hemolytic effects. Hemolytic Peptide Identification Server has also been used to design non-toxic agents for use in aquaculture. RY12WY, a high thermostable peptide, was synthesized, and its hemolytic potency was evaluated. Its low hemolytic tendency is consistent with virtual analysis [157]. Despite most successful cases, some incorrect predictions have been reported. HemoPred classified Enterocins K1 and EJ97 as hemolytic and non-hemolytic, respectively. However, through a traditional evaluation of hemolytic nature, Reinseth and colleagues demonstrated that both *Enterococcus* spp. bacteriocins are non-hemolytic peptides [158]. Insignificant erythrocyte lysis was observed up to a concentration of 1 mg/mL. Taken together, these findings underline the usefulness of computational technologies in peptide drug discovery. New peptide sequences, experimental validations, and updated databases should improve the reliability of the results.

## 5. Case Study

The high performance of hemolytic activity methods enables accurate screening of non-toxic peptides. The use, evaluation, and refinement of models are key to new advances in computational peptidology. In this direction, to further explore the model performances, we predicted the hemolytic activity of peptides using seven of the computational methods presented in Table 3. Seven data sets based on HemoPI-1 (main/validation), HemoPI-2 (main/validation), HemoPI-3 (main/validation) and HAPPENN were employed to benchmark the main models. These data sets were chosen to represent the diversity and criteria used to generate the virtual screening tools. The first data set consists of peptides with hemolytic action or lack thereof. The second is formed by high hemolytic and non-hemolytic peptide sequences. Similarly, HemoPI-3 discriminates highly and poorly hemolytic peptides. The last data set, HAPPENN, is composed of both hemolytic and non-hemolytic peptides, including sequences with *N*-terminal acetylation and *C*-terminal amidation. HAPPENN is recognized as a high-quality and well-established data set [113].

Our initial assessment represents a closer look into state-of-the-art prediction accuracy for the hemolytic nature classification task. However, future analyses are required to elucidate possible biases, as well as the standardization of the classification of non-hemolytic and hemolytic peptides used for the construction of data sets. Some of them include peptides with low hemolytic as non-hemolytic activity. In two-layer prediction frameworks such as HLPpred-Fuse, these peptides are classified as low-intensity hemolytic [35]. The concentration limits between non-hemolytic and low hemolytic activity differ significantly in some studies [36]. In our analysis, non-hemolytic and poor hemolytic peptides were considered as negative examples. Motivated by these considerations, HLPpred-Fuse was challenged considering the second-layer prediction (low/high) results, except for analysis of HemoPI-1 data sets. In this case, the first-layer prediction results were used.

Only peptides consisting of 7–35 residues in length and made of natural amino acids were selected for our bioinformatics screening. In brief, this approach produced seven data sets: HemoPI-1 7–35main, HemoPI-1 7–35val, HemoPI-2 7–35main, HemoPI-2 7–35val, HemoPI-3 7–35main, HemoPI-3 7–35val, and HAPPENN 7–35, which are composed by 846, 207, 765, 190, 1175, 294, and 1547 sequences, respectively. Details of the composition (positive and negative) of each data set are summarized in Supplementary Table S1. All data is freely available on GitHub at https://github.com/albert-robles1101/hemolytic-prediction-of--peptides,accessed on 25 January 2022. The screening was repeated five times, and the classification was assigned according to most predictions. (0: non-hemolytic and 1: hemolytic). Divergent analyzes were observed in HemoPred for some peptide sequences. Four widely used metrics, including Acc, sensitivity (SN), specificity (SP), and MCC, were adopted to evaluate the performance of selected models. These statistical parameters were calculated as follows:

(2) $$\;\mathrm{Acc}=\frac{\mathrm{TP}+\mathrm{TN}}{\mathrm{TP}+\mathrm{TN}+\mathrm{FP}+\mathrm{FN}}\times 100$$

(3) $$\mathrm{Sn}=\frac{\mathrm{TP}}{\mathrm{TP}+\mathrm{FN}}\times 100$$

(4) $$\;\mathrm{Sp}=\frac{\mathrm{TN}}{\mathrm{TN}+\mathrm{FP}}\times 100$$

(5) $$\;\mathrm{MCC}=\frac{\mathrm{TP}\;\times \;\mathrm{TN}-\mathrm{FP}\;\times \;\mathrm{FN}}{\sqrt{\left(\mathrm{TP}+\mathrm{FP}\right)\left(\mathrm{TP}+\mathrm{FN}\right)\left(\mathrm{TN}+\mathrm{FP}\right)\left(\mathrm{TN}+\mathrm{FN}\right)}}\times 100\;$$

where TP, TN, FP, and FN stand for the number of true positives, true negatives, false positives, and false negatives, respectively.

Table 4 summarizes the prediction results of all seven high-throughput computational methods on the test data sets. In general, the models were able to distinguish hemolytic and non-hemolytic sequences. In some cases, best-in-class performance was achieved. Each program showed an Acc higher than 90% for at least one of the data sets. Most Acc and MCC values are in the ranges previously determined by the developers (Acc: 76.0%–98.4% and MCC 0.55–0.97). Some models, such as HemoPI-1 and Plisson models, showed low Acc and MCC values when challenged by HemoPI-2 (main/val) HemoPI-3 (main/val), and HAPPENN data sets. Our findings are in agreement with previous analysis reporting the better discriminative power on the hemolytic effect of peptides of HAPPENN [36] and HLPpred-Fuse [35] models than the HemoPi-1 server [26], particularly when they were applied to estimate the activity of peptide sequences derived from HemoPI-2 and HemoPI-3 data sets.

Since the models were not trained under the same conditions and data sets, a direct comparison is not completely adequate. This is a simple case study for an initial assessment to encourage the application of high-throughput screening of peptide libraries. The performance metrics determined for some predictors can be overestimated due to the similarity of some benchmark data sets and the peptide sequence data previously used in optimizing the hyperparameters of these computational methods. Because of that, future studies should consider different and large data sets. Additionally, for a fair comparison, the specifications of each model and their training data must be thoroughly explored and merged. However, collectively, our large-scale analysis confirms the applicability and robustness of virtual tools and their contributions to a sustainable and cost-effective design and discovery of non-toxic peptides.

## 6. Current Strategies to Improve the Selectivity Index of Therapeutic Peptides

**Figure 4:**
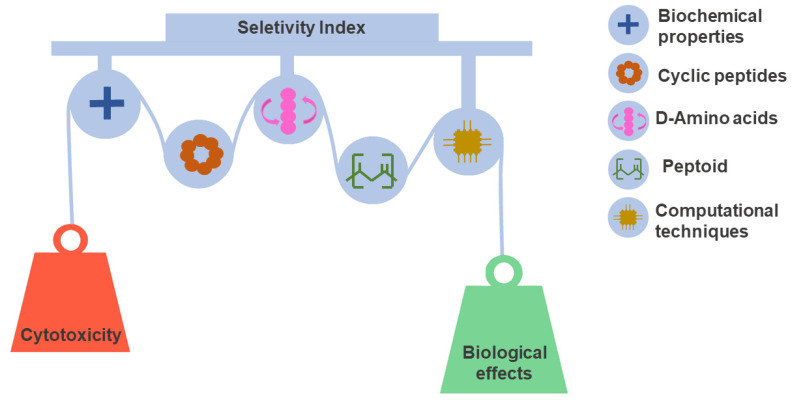
Peptide selectivity optimization strategies. Selectivity is a favorable characteristic of considerable significance for the success rate of drug candidates. However, selectivity optimization is a complex task that must balance the properties that govern toxic and therapeutic effects. Different design and synthesis strategies have contributed to this objective. In the design step, the evaluation of physicochemical properties and SAR relationships integrated with computational techniques play a decisive role in peptide selection. In the same context, cyclization, use of D amino acids, and peptidomimetics have been key for the development of stable and selective peptides.

Tuning selectivity is a daunting task on the path toward developing peptide-based drugs. Determining peptide selectivity is a mandatory requirement during preclinical research [159,160]. As discussed above, selectivity, quantified by the SI, results from a fine-tuned fit between toxicity and biological effect. This delicate balance remains a challenge explored in many SAR studies [161,162]. For this reason, we discuss and illustrate the leading design and synthesis strategies used to reduce toxicity and increase the potency of peptides. Figure 4 shows approaches that have been useful in this regard.

### 6.1. Optimization and Complementation of the Physicochemical Properties

Physicochemical properties such as positive net charge and amphipathicity significantly influence the bioactivity of most AMPs (mainly reported as α-helical structures) [163,164]. The net positive charge is related to the initial electrostatic interaction with anionic phospholipids and lipopolysaccharides that make up the membranes of certain pathogens [165]. On the other hand, mammalian cells such as RBCs have zwitterionic phospholipids in the outer leaflet of their membranes, which are not as much affected by the positive charge as they can be by the hydrophobic properties of the peptide [159]. Highly hemolytic peptides interact with phosphatidylcholine, an abundant component of zwitterionic membranes [166]. In contrast, the cholesterol in mammalian membranes inhibits peptide binding [167].

The chemical (solid-phase) peptide synthesis presents versatility and accessibility to generate several analogs of the same parental molecule, which is very useful for determining the residues and regions responsible for the biological activity of interest. Some examples of increased selectivity, and therefore, a decreased hemolytic activity due to modifications in the physicochemical properties of analogous peptides, have been observed for peptides such as: peptides derived from leucine zipper [109], magainins [106], hybrids of the melittin [168], PMAP-36-derived peptides [169] and clavaspirin [170].

Not all the structural parameters of a peptide are independent, so it is challenging to determine what characteristics can significantly influence selectivity [159]. In many studies, reducing the peptide hydrophobicity might lead to an unintended decrease in antimicrobial activity [171]. In another case, the substitution of a single hydrophobic residue for a positively charged residue has reduced hemolytic activity without compromising antimicrobial activity [172].

There is also evidence that the interchange of amino acids with similar chemical properties can affect peptide activity. Four phenylalanine residues of the cathelicidin-BF15-a1 peptide were replaced by tryptophan residues, which resulted in increased activity against *E. coli* (MIC reduction from 9.6 to 2.1 µM) and *Bacillus subtilis* (MIC reduction from 38.7 to 4.3 µM). The low hemolytic activity was not affected by this modification (HC_50_ > 320 µg/mL in both cases) [173]. In order to maintain the native primary structure properties, one can choose to carry out end modifications such as *C*-amidation or *N*-acetylation, which could increase the activity of the molecule and its stability within an organism [25]. Conjugations with nanoparticles are another interesting strategy that has diminished toxicity and increased biological activity [174].

### 6.2. Cyclization

Cyclization has been an adequate tool in peptide science, mainly to increase peptide stability and constrain a three-dimensional structure to enhance its desired biological effect [159,175]. For instance, the cyclization has prevented the hydrophobic region of a cyclized melittin analog from being altered, retaining antimicrobial capacity while reducing hemolytic activity [176]. However, such a process is sequence dependent, and a case-by-case analysis is required. For example, the cyclization of a magainin analog significantly reduced both its antimicrobial and hemolytic activities [176]. In contrast, the cyclization of the RRWWRF peptide increased both its hemolytic and antimicrobial activities [106].

### 6.3. Incorporation of D Amino Acids

Peptide selectivity can also be improved by gaining proteolytic resistance. The peptide lifetime in the body is extended, reaching the same therapeutic effects at lower peptide doses and reducing toxic effects [159]. The incorporation of amino acids in the D configuration confers resistance to the action of proteolytic enzymes, which cannot exert their actions due to steric hindrances [177,178]. This has allowed the evaluation of some peptides on an in vivo scale, obtaining highly relevant results [179,180]. Examples of this type of modification with favorable results in selectivity include melittin and other peptides composed mainly of leucine and lysine [181,182]. Additionally, incorporation of D amino acids in the sequence of an AMP may further alter its amphipathic structure in such a way that peptide hydrophobic interactions with the zwitterionic host cell membranes, and consequently hemolysis, can be substantially reduced [183].

### 6.4. Use of Peptoids

Another way to disturb the amphipathic α-helices in certain AMPs is the use of peptoids, i.e., peptide analogs comprising *N*-substituted glycines, i.e., amino acid residues where side chains are linked to the nitrogen rather than to the α-carbon; consequently, amide groups in peptoids are unable to act as hydrogen bond donors [166]. An example of this approach concerns the Leu/Lys-rich KLW peptide, in which introduction of *N*-substituted glycines at positions 9 and 13 decreased the hemolytic activity (0% hemolysis up to a 100 µM peptide concentration) while enhancing the antimicrobial action from minimal inhibitory concentrations (MIC) of 4 to 8 µM (native sequence) to 1 to 4 µM (peptoid analog) against several bacteria [184]. The same strategy was applied to other peptides such as melittin [185] or cathelicidins [186] to obtain better selectivity.

### 6.5. Bioinformatics Tools

Although chemical synthesis is essential to the development of new peptide candidates, each synthetic process implies a considerable investment in the human workforce, techniques, and resources [187,188]. The use of alternative approaches, including machine learning tools, has risen in recent years [189]. Such tools greatly reduce costs, as their outputs direct laboratory resources and efforts in a most cost-effective manner, through estimations, predictions, and comparisons of relevant properties based on available information and adequate prediction models [72]. Many computational tools that are complementary to toxicity predictors promise to accelerate the development of bioactive peptides, as follows.

The investigation carried out by Kamech et al. [92] is one emblematic example of the relevance of bioinformatics toward the development of peptide therapeutics. These authors have developed software named Mutator, which creates specific sequence substitutions that enhance the selectivity and effectiveness of peptides. This resource generated analogs of the native peptides XT-7 and ascaphin-8, which were later synthesized and evaluated in vitro. According to Mutator, the suggested substitutions should raise the SI from <37 to >80. Experimentally, SI > 130 were found for *S. aureus* and *E. coli*. Moreover, the mutant peptide derived from XT-7 displayed an SI that was increased from 5 to >270 on *Pseudomonas* *aeruginosa*. Mutator has produced data on 26 peptides with potent antimicrobial activity and SI values > 20. Note that the training data were predominantly linear α-helical amphipathic peptide sequences that adopt an α-helical amphipathic structure; hence, the Mutator has so far shown its limitations to molecules with these structural characteristics.

Machine learning (ML) is another important and very useful tool for the generation of novel peptide sequences [190]. For example, Capecchi et al. [38], developed a generative model using RNN and the DBAASP database that revealed 28 new peptides of 15 residues in length at best that differed in point mutations with the data from the training set. Of the 28 sequences, 8 peptides were recognized as non-hemolytic and likely to be active against different bacteria strains. Another example of a tool based on ML and focused on predicting AMP sequences is AMAP [191]. This tool has the ability to use a multilevel classification that allows predicting 14 different types of biological activities for a given peptide. The value generated is correlated to the possible antibacterial effectiveness. AMAP was evaluated with the proof-of-concept peptide P276, which has a powerful antimicrobial activity. The tool cataloged it precisely as a promising AMP candidate (AMAP score = 1.70), another similar server (MLAMP) classified it as a non-antimicrobial peptide.

The selectivity of specific peptides toward their target cells can be assured by a few predictive models, as outlined by Li et al. [192]. These authors aimed at identifying the characteristics and factors having a broad influence on peptide selectivity by means of an RF algorithm that correlates the properties of the peptide sequences with their biological activity. The models generated yielded high precision predictions, and their interpretation indicated that selectivity is mediated by a strong relationship between properties related to solubility and charge.

Antimicrobial activity is not the only biological function affected by typical AMPs since their structural and functional diversity enables them to act on other lipid membranes, such as those of cancer cells. In this context, Gabernet et al. [193] developed an ML model that discriminated peptides with or without anticancer activity. The predictive model was experimentally validated through the synthesis and biological evaluation of 12 model peptides, revealing 83% of successful predictions. A design algorithm, known as simulated molecular evolution, was also used that increased the selectivity of the peptides by 5 times with respect to human endothelial cells and by 10 times with respect to RBCs, illustrating the benefits of using ML-guided design and optimization for peptide-based drugs.

## 7. Future Perspectives and Concluding Remarks

Accurate computational predictions are extremely attractive in the early stages of drug discovery and are revolutionizing the development of peptide-based therapeutics. Bioinformatics tools constitute a safe gateway that gives valuable hints on which therapeutic peptides are worthy of being progressed while preventing the advancement of likely toxic molecules. Toxicity remains a major hurdle toward the clinical translation of peptides, and standard approaches to avoid it are time and resources consuming, often running in the opposite direction of the 3Rs guidelines for animal experimentation. In silico tools have provoked a boom in the ability to predict peptides as hemolytic or non-hemolytic due to the accessibility and abundance of DBs, enabling the understanding and systematization of structural determinants and key properties underlying peptides’ hemolytic effects. These DBs are essential for the construction of models capable of classifying a vast number of peptide sequences and guiding de novo peptide design. Currently, 12 tools are freely available, 10 of which specifically address peptides’ hemolytic action. Based on the metrics evaluated, our findings support that these virtual tools are of great use for the scientific community. The relatively high reliability and resolving power open new avenues for the design and development of prospective clinical peptide drugs with minimal cost, time, and resources. The experimental validation of these predictions is necessary and should contribute to the refinement of the models. A comparison of the predictive results obtained using the current methods to evaluate mega data of peptide databases is a promising source of valuable clues for the improvement and accuracy of high-quality computational technologies. Future studies should assess the sampling bias within available models, which may be influenced by structures, sequence diversity, and amino acid composition. The transition to quantitative analysis, such as the development of regression models to predict HC_50_ values, is also of great relevance and usefulness for in silico peptide design. Finally, innovative programs must consider predicting the balance between toxicity and therapeutic effect, i.e., selectivity. Thus, the future design of peptide pharmaceuticals should be greatly favored by the interplay between computational, in vitro, and in vivo approaches.

## Figures and Tables

**Table 1:** Hemolytic activity and selectivity indices for reference antimicrobial peptides. A promising peptide drug candidate must combine low or no toxicity and high biological activity. Examples include highly active peptides evaluated against *Staphylococcus aureus* and *Escherichia coli*.

Peptide	Microorganism	HC_50_ (µM)	SI	Ref *
[K^19^] ascaphin-8	*E. coli*	>800	>160	[92]
[I^2^, K^19^] ascaphin-8	*S. aureus*	>800	>170	[92]
I16-A piscidin-1	*S. aureus* ATCC 25923	500	>200	[94]
[K^2^, K^16^] XT-7	*S. aureus*	>800	>267	[92]
DiPGLa-H	*S. aureus* ATCC 29213	270	360	[95]
Adepantin-2	*E. coli*	400	400	[96]
Kiadin-1	*E. coli* ATCC 25922	340	450	[95]
[I^2^, K^19^] ascaphin-8	*E. coli*	>800	>480	[92]
Hymenochirin-10B	*E. coli*	>512	>512	[97]
Dadapin-1	*S. aureus* ATCC 29213	670	670	[98]
Flexampin	*E. coli*	>200	670	[99]
Papiliocin	*E. coli*	200	800	[100]

* Ref: Reference.

**Table 2:** Databases on antimicrobial peptides (AMPs) and hemolytic peptides (HLPs). Databases (DBs) are crucial starting points for identifying patterns and developing predictive methods. This table details the main DBs that bring together a large amount of information relevant to the strengthening and development of peptide science, especially computational peptidology. Specifically, in hemolysis prediction tools, peptide DBs detailing hemolytic activity are indispensable for the development of classification models. For their part, DBs on AMPs are equally useful to guide the next steps of evaluation of the potential of peptides. DBs were accessed on 3 March 2022 to confirm the number of peptides.

Antimicrobial Peptides Databases				
Name	Year	#Peptides	Description	Reference
DBAASP	2021	18719	Manually curated DB on peptides’ SAR	[139]
LAMP2	2020	23253	Links AMPs from 16 different DBs	[22]
DRAMP 2.0	2019	22259	Covering sequence, structure, activity, and physicochemical features, as well patents, clinical and other reference information on AMPs	[140]
InverPep	2017	774	AMPs from invertebrates	[141]
APD3	2016	3324	Natural peptides with knownsequences and activities	[142]
CAMPR3	2016	8164	Covering sequences, structures, and family-specific signatures of AMPs, including their biological sources and target organisms	[143]
BaAMPs	2015	221	AMPs with specific anti-biofilm activity	[144]
ParaPep	2014	519	Focused on anti-parasitic peptides	[145]
AVPdb	2014	2683	Focused on antiviral peptides	[146]
YADAMP	2013	2525	Focused on antibacterial peptides	[147]
MilkAMP	2013	371	Focused on AMPs from milk	[148]
DADP	2012	1923	Focused on AMPs from the Anura family	[149]
BACTIBASE	2010	230	Focused on bacteriocins	[150]
PhytAMP	2009	271	Focused on AMPs from plants	[151]
**Hemolytic/Toxic Peptides Databases**				
ToxinPred	2013	1805	Focused on small toxins	[40]
Hemolytik	2014	2000	Covering complete information on the origin, hemolytic activity, reported function, structural properties (chirality, linear versus cyclic backbone, etc.), and existing modifications, if any	[138]
HemoPiMOD	2020	1176	Focused on chemically modified HLPs	[34]
DBAASP-Hemo *	2021	2262	A filtered sub-DB of DBAASP that collects peptides’ activity specifically against *P. aeruginosa*, *A. baumannii*, and *S. aureus* and RBCs. In total, it contains 1319 HLP and 943 non-HLP	[38]

* Data set generated by Capecchi et al. [38] and identified here as DBAASP-Hemo.

**Table 3:** Accuracy, Matthew’s correlation coefficient, summary of data set, and evaluation strategies of the models used by each of the ten programs used to predict the hemolytic activity of peptides. Accuracy and MCC are only reference values and are not comparable between different models unless they have used the same database. HemoPiMod is the only hemolytic action prediction server that takes into account chemical modifications of peptides. HemoNet is the only server that focuses on C/N terminal and L or D amino acid modifications to perform classification. All other tools predict hemolytic activity from natural sequences without any modification.

Tool	Model	Data Set *	Training-Testing	Validation	Acc	MCC	Web Server **	Ref
HemoPImod	RF	583/583	80%–20%	5-fold	78.33	0.56	√	[34]
HAPPENN	NN	1543/2195	75%–8.3%	10-fold and external test	85.70	0.71	√	[36]
Plisson et al. model 1	GBoost	552/552	80%–20%	Stratified 10-fold and external test	96.50	0.93	NA	[37]
Plisson et al. model 2	LDA				95.10	0.90		
Plisson et al. model 3	XGBoost				95.70	0.91		
ToxinPred ***	SVM	Main: 1805/3593 Alternative: 1805/12541	Independent test	5-fold and 10-fold	94.50	0.88	√	[40]
HemoPred	RF	HemoPI-1: 552/552 HemoPI-2: 552/462 HemoPI-3: 885/738	HemoPI-1: 80–20% HemoPI-2: 80.2–19.8% HemoPI-3: 80–20%	5-fold and external test	95.90	0.92	√	[33]
HemoPI-1	SVM hybrid	552/552	80–20%	5-fold	95.30	0.91	√	[26]
HLPpred-Fuse	ERT	First layer: Training: 433/423 First independent: 666/1999 Second layer: Training: 671(high)/423(low) Second independent: 168(high)/147(low)	First layer: 24.3–75.7% Second layer: 77.6–22.4%	10-fold, independent test and case study	98.40	0.97	√	[35]
HemoNet	SNN	2056/2881	20–80%	5-fold, external data set, non-redundant cross-validation	Nd	0.55	NA	[113]
Capecchi et al.	RNN	1319/943	75–25%	Test set	76.00	0.52	NA	[38]
ATSE ***	NN	1932/1932	85–15%	10-fold	95.20	0.90	√	[39]

* Data set: ratio of toxic/non-toxic or hemolytic/non-hemolytic peptides. ** Web server (accessed on 25 January, 2022): HemoPImod: https://webs.iiitd.edu.in/raghava/hemopimod/index.html, accessed on 25 January 2022; HAPPENN: https://research.timmons.eu/happenn, accessed on 25 January 2022; ToxinPred: https://webs.iiitd.edu.in/raghava/toxinpred/index.html, accessed on 25 January 2022; HemoPred: http://codes.bio/hemopred/, accessed on 25 January 2022; HemoPI-1: https://webs.iiitd.edu.in/raghava/hemopi/batch.php, accessed on 25 January 2022; HLPpred-Fuse: http://thegleelab.org/HLPpred-Fuse/index.html, accessed on 25 January 2022; ATSE: http://server.malab.cn/ATSE, accessed on 25 January 2022; *** Model that predicts the peptide toxicity. √: Available. NA: Non-available. Nd: Non-determined.

**Table 4:** Test results for state-of-the-art predictors based on seven data sets. HemoPI-1 7–35main, HemoPI-1 7–35val, HemoPI-2 7–35main, HemoPI-2 7–35val, HemoPI-3 7–35main, HemoPI-3 7–35val, and HAPPENN 7–35 data sets were employed to challenge the main predictors.

Method	Data Set	Acc	Sn	Sp	MCC
HAPPENN	HemoPI-1 7–35main	82.03	69.10	95.02	0.66
	HemoPI-1 7–35val	80.68	65.38	96.12	0.65
	HemoPI-2 7–35main	75.69	69.10	83.87	0.53
	HemoPI-2 7–35val	77.89	65.38	93.02	0.60
	HemoPI-3 7–35main	85.79	79.47	93.42	0.73
	HemoPI-3 7–35val	85.71	81.88	90.30	0.72
	HAPPENN 7–35	96.51	96.19	96.70	0.93
Plisson et al. (2020), model 1	HemoPI-1 7–35main	95.74	94.10	97.39	0.92
	HemoPI-1 7–35val	95.65	91.35	100.0	0.92
	HemoPI-2 7–35main	64.05	94.10	26.69	0.29
	HemoPI-2 7–35val	61.58	91.35	25.58	0.23
	HemoPI-3 7–35main	58.64	87.25	24.06	0.15
	HemoPI-3 7–35val	62.24	91.87	26.87	0.25
	HAPPENN 7–35	57.85	90.99	38.14	0.32
Plisson et al. (2020), model 2	HemoPI-1 7–35main	100.0	100.0	100.0	1.00
	HemoPI-1 7–35val	95.65	95.19	96.12	0.91
	HemoPI-2 7–35main	62.75	100.0	16.42	0.31
	HemoPI-2 7–35val	56.84	95.19	10.46	0.11
	HemoPI-3 7–35main	59.32	95.18	15.98	0.19
	HemoPI-3 7–35val	59.86	96.25	16.42	0.21
	HAPPENN 7–35	50.42	93.93	24.54	0.23
Plisson et al. (2020), model 3	HemoPI-1 7–35main	99.88	99.76	100.0	0.99
	HemoPI-1 7–35val	96.62	94.23	99.03	0.93
	HemoPI-2 7–35main	64.31	99.76	20.23	0.34
	HemoPI-2 7–35val	57.89	94.23	13.95	0.14
	HemoPI-3 7–35main	59.91	93.00	19.92	0.19
	HemoPI-3 7–35val	59.86	95.00	17.91	0.21
	HAPPENN 7–35	51.91	91.85	28.14	0.24
HemoPred	HemoPI-1 7–35main	80.14	86.79	73.46	0.61
	HemoPI-1 7–35val	78.26	84.62	71.84	0.57
	HemoPI-2 7–35main	85.23	86.08	84.16	0.70
	HemoPI-2 7–35val	86.84	84.62	89.53	0.74
	HemoPI-3 7–35main	97.53	97.98	96.99	0.95
	HemoPI-3 7–35val	96.94	97.50	96.27	0.94
	HAPPENN 7–35	80.48	94.11	72.37	0.64
HemoPI-1	HemoPI-1 7–35main	97.75	98.11	97.39	0.96
	HemoPI-1 7–35val	97.58	98.08	97.09	0.95
	HemoPI-2 7–35main	61.96	98.11	17.01	0.27
	HemoPI-2 7–35val	58.42	98.08	10.47	0.18
	HemoPI-3 7–35main	59.15	95.18	15.60	0.18
	HemoPI-3 7–35val	60.20	96.25	17.16	0.22
	HAPPENN 7–35	50.42	94.28	24.33	0.24
HLPpred-Fuse	HemoPI-1 7–35main	99.88	99.76	100.0	0.99
	HemoPI-1 7–35val	98.55	97.12	100.0	0.97
	HemoPI-2 7–35main	83.14	83.02	83.28	0.66
	HemoPI-2 7–35val	82.11	76.92	88.37	0.65
	HemoPI-3 7–35main	96.85	95.02	99.06	0.94
	HemoPI-3 7–35val	80.61	82.50	78.36	0.61
	HAPPENN 7–35	75.89	80.24	73.30	0.52

## Data Availability

Publicly available datasets were analyzed on this study. This data can be found here: https://github.com/albert-robles1101/hemolytic-prediction-of--peptides (accessed on 24 January 2022).

## Supplementary Materials

The following supporting information can be downloaded at: https://www.mdpi.com/article/10.3390/ph15030323/s1. Table S1: The size and composition of data sets.
