# Supplementary material for: Traditional and Computational Screening of Non-Toxic Peptides and Approaches to Improving Selectivity
Source: Pharmaceuticals (Basel). 2022 Mar 8;15(3):323. doi: 10.3390/ph15030323 (PMC8953747; doi:10.3390/ph15030323)
Supplement: Supplementary file 1 [file pharmaceuticals-15-00323-s001.zip › pharmaceuticals-1587070-supplementary.pdf]

pharmaceuticals

Table S1. The size and composition of data sets.

Datasets	Size (Peptides)	Classification	Based on
**HemoPI-1 7-35main**	846	424 positives	HemoPI-1 main
		422 negatives	
**HemoPI-1 7-35val**	207	104 positives	HemoPI-1 validation
		103 negatives	
**HemoPI-2 7-35main**	765	424positives	HemoPI-2 main
		341 negatives	
**HemoPI-2 7-35val**	190	104 positives	HemoPI-2 validation
		86 negatives	
**HemoPI-3 7-35main**	1175	643 positives	HemoPI-3 main
		532 negatives	
**HemoPI-3 7-35val**	294	160 positives	HemoPI-3 validation
		134 negatives	
**HAPPENN 7-35**	1547	577 positives	HAPPENN
		970 negatives	
